# Formulation, development and characterization of a novel functional fruit snack based on fig (*Ficus carica L*.) coated with sugar-free chocolate

**DOI:** 10.1016/j.heliyon.2020.e04350

**Published:** 2020-07-05

**Authors:** Samira Yeganehzad, Maryam Kiumarsi, Narjes Nadali, Mansour Rabie Ashkezary

**Affiliations:** a Department of Food Processing, Research Institute of Food Science and Technology (RIFST), Mashhad, Iran; b Department of Agricultural, Food and Forest Sciences (SAAF), University of Palermo, Viale delle Scienze, 90128 Palermo, Italy

**Keywords:** Food science, Food technology, Fruit snack, Fig (*Ficus carica L*.) powder, Chocolate, Persian gum, Xanthan gum

## Abstract

The aim of the present investigation was to explore the possibility of developing a fruit snack formulation based on dried fig powder and chocolate-coated. Dried Fig (*Ficus carica L*.) powder with a maximum particle size of 354 μm and the lowest compaction force was formulated as the core. Persian gum was prepared at the concentrations of 1.5, 2 and 2.5% and xanthan gum was prepared at the levels of 0.25, 0.39 and 0.54% as the middle layer to the coating of the core. Regarding rheological assessments, sugar-free chocolate containing 29.3% isomalt was selected for the coating of the outer chocolate shell in the entitled snack. Textural analysis showed that coating of the core with hydrocolloids decreased hardness and adhesiveness of the samples (*p* < 0.05). It was also observed that increasing the xanthan gum and Persian gum concentration led to the reduction of adhesiveness in the snacks (*p* < 0.05). Coating of cores with hydrocolloids resulted in lower thickness of the chocolate outer shell, as well (*p* < 0.05). Results of the sensory evaluation tests demonstrated that, the samples with hydrocolloid coating were the most preferred ones by the panelists.

## 1 Introduction

The development of novel snacks with nutritious ingredients has an effective role in improving the diet quality. Eating between the main meals or snacking is a popular behavior throughout the world, which is more appreciated by children (Adams and Savage, 2017). Approximately 25% of daily energy intake is obtained from the snack consumption. Therefore, its nutritional quality has to be highly considered (Green et al., 2017).

Consumption of fruit-based snacks increases the intake of nutrients and phytochemicals which leads to the positive health effects and could help people to gain the recommended intake (Potter et al., 2013; Roe et al., 2013). Fruit snacks may be prepared as dragée comprised of a core portion coated with an outer shell. A wide range of edible ingredients could be used as cores and coatings. Nowadays, natural ingredients such as fruits have gained interest as a suitable material for core formulation (Beckett, 2009). *Ficus carica L*. is one of the oldest cultivated fruit trees, belonging to the Moraceae family, commonly grown in subtropical regions. Fig can be considered as an excellent source of dietary fiber, minerals and vitamins. These include iron, calcium, potassium, thiamin (B1), riboflavin (B2), and lignin. The high amount of soluble fiber in fig plays an important role in regulating the blood sugar as well as weight loss regulation (Solomon et al., 2006). The fresh fruit's polyphenol and flavonoid compounds provide high antioxidant capacity in total (Yang et al., 2009). In addition, it is worth knowing that dried figs have higher antioxidant capacity when compared fresh figs (Arvaniti et al., 2019). Moreover, fig is used in traditional medicine due to its health promoting effects on constipation, cardiovascular diseases, inflammation and spasmodic problems (Oliveira et al., 2009).

Raising individual awareness about the disadvantages of sugar overconsumption on human health has led to the production of low or sugar-free products formulated with sugar alternatives. Isomalt is a non-hygroscopic alcoholic sugar with a mild sweetness (40% sucrose) and sugar-like physical properties. This sugar is a suitable sweetener in development of confectionary products that are low glycemic, low insulin-emic and the ones that promote dental health (van Loveren et al., 2012). Emulsifiers are surface active agents which improve the rheological and textural properties of chocolate. They could also reduce the fat required, to make chocolate while maintaining the desirable flow properties (Afoakwa et al., 2007). Therefore, soybean lecithin as a mixture of natural phosphoglycerides is widely used in chocolate manufacture.

Different hydrocolloids such as Arabic gum, xanthan, Persian gum, gum tragacanth, gelatin and arboxymethylcellulose could be applied as the middle layer coating between the core and shell; in order to stop the migration of moisture, aroma and lipid from the core. Gum coating creates a smooth and polished surface around the core and leads to a uniform chocolate distribution (Beckett, 2009). Persian gum (local name: Zedo gum) is an exudate of the almond tree which is famous to grow naturally in Ian. It is well known for its traditional health effects. Thirty percent (w/w) of Persian gum can be easily dissolves in water. The gum's dispersion tolerates wide range of pH and heat treatment. Persian gum has been applied in gelatin-free confectionery; for texture modification in dairy formulations and bakery products and as coating in deep fat frying to reduce oil absorption. Other emulsifying and stabilizing roles were summed up in a review paper (Abbasi, 2017). Persian gum can properly compete with available industrial gums, due to its working properties. Xanthan is an industrial microbial polysaccharide, which is produced by *Xanthomonas campestris*. Despite its high molecular weight, xanthan gum dissolves easily in both cold and hot water. Its ability to form a high viscosity solution at low concentrations and its stability regarding a wide range of varieties such as salt concentration, temperature and pH, has made xanthan an appropriate ingredient in food products (Garcıa-Ochoa et al., 2000).

The main goals of this study were: 1) To develop a formulation for the fig fruit snack 2) To create and appealing appearance for the snack with appropriate sugar-free chocolate as outer shell 3) To improve sensory and textural properties of the snack by the application of Persian gum and xanthan gum solution as the middle layer.

## 2 Materials and methods

### 2.1 Preparation of the core

Fig fruits (*Ficus carica L*.) were purchased from a research botanical garden in Iran. The figs were washed and then dried in a vacuum oven at 70 °C for 48 h. Powder was obtained by grinding the dried figs. The powders were sifted through three sieves with mesh sizes of 707, 505, 354 μm and shaped by a manual machine to form the convex core with 1 cm in diameter and it was considered as the core of the snack.

#### 2.1.1 Fig powders properties

Water activity of the fig powders were measured using water activity analyzer at 25 °C (Lab Master Standard, Novasina, Swiss). 1 g of the powder sample was mixed with 10 ml distilled water and pH measurement was carried out using a digital calibrated pH-meter at 25 °C (Metrohm, Swiss). The moisture and ash content were performed based on approved procedure AACC, NO 44-16 and 08-01, respectively. Sticky point temperature was determined by means of rotational viscometer, spindle sc4 (Brookfield) according to the procedure described by Özkan et al. (2002) with some modification. Sample (6 g) was placed in a cup with a temperature-controlled jacket. The sample was allowed to equilibrate to a certain temperature for 30 min. Temperature increased with the rate of 1°C/3 min and the torque measurement was carried out at the speed of 45 rpm. The sticky-point temperature was determined as a point where a sharp increase in torque occurred. The powder compactibility was measured by texture analyzer (TA.XTPlus, Stable Micro System, England). The powder was deposited into the sample container and a compression test was performed. For this experiment, the compaction rig probe was used with 1 mm s^−1^ test speed. The force (F) required for 60% compaction of powder was recorded.

#### 2.1.2 Preparation of hydrocolloid coating

Persian gum and xanthan gum were purchased from Parsian Gum Tam Co. (Mashhad, Iran). Rotational viscometer (Brookfield) equipped with ULA spindle and water controlling system (Julabo, Japan) was used to study the rheological behavior of samples. The shear stress was plotted against shear rate in a range of 0.1–100/s to determine the appropriate concentrations based on the equal apparent viscosity for both gums. Persian gum and xanthan gum at the concentrations of 1.5, 2, 2.5% and 0.25, 0.39, 0.54% (w/v) were selected, respectively. Gum solutions were prepared and hydrated overnight, one the day before using.

### 2.2 Preparation of chocolate coating

The main materials required for the chocolate production were as cocoa powder (12% fat, Cargill, Denmark), cocoa butter (Cargill, Denmark), lecithin (Palsgaard Company, Denmark) and isomalt (Beneo Company, Germany). Four formulations of sugar-free chocolates were produced (Table 1). The raw materials for chocolate production were weighted and then poured into a laboratory ball mill (Armankherad Company, Mashhad, Iran). The mixing, milling and particle size reduction and conching were carried out at 45 °C in 100 rpm. The samples were tempered before use.

**Table 1:** Formulation of sugar-free chocolates.

Sample code	Isomalt (%)	Lecithin (%)	Cocoa butter (%)	Cocoa powder (%)
A	34.5	0.5	35	30
B	34.3	0.7	35	30
C	29.5	0.5	40	30
D	29.3	0.7	40	30

### 2.3 Particle size distribution of chocolate

Particle size distribution of prepared chocolates were determined using particle size analyzer (Sald 2101, Shimadzu, Japan). Chocolates were dispersed using hexane and were then vigorously stirred under ultrasonic waves (50 Hz, 200 W) for 5 min. After initial preparation, samples were transferred to the laser chamber. The parameter of the largest particle size (D_90_) was reported in a micrometer scale.

### 2.4 Rheological properties of chocolate

Rheological properties of the molten chocolates were carried out using a stress-controlled rheometer (Rheolab Qc, CTD60 Instruments, Germany) with concentric cylinder system (cup: 28.92 mm and bob: 26:66 mm) as described by Rabie Ashkezary et al. (2017). Each chocolate sample was prepared by heating in an incubator at 50 °C for 75 min. After melting, samples were weighed into the cup and measurements were performed using the official method for chocolate (ICA, 2000). The samples were pre-sheared at 5 s^−1^ rate for 15 min at 40 °C, before the measurement cycles started. Shear stress was measured as a function of increasing shear rate from 2 s^−1^ to 50 s^−1^ (ramp up) within 2 min, holding at 50 s^−1^ for 60 s, then decreasing from 50 s^−1^ to 2 s^−1^ (ramp down) within 2 min. Temperature was fixed at 40 ± 0.1 °C during the process. Regarding different non-Newtonian models, the data were best fitted to the Casson model (R^2^ > 0.98). Therefore Casson yield value and Casson plastic viscosity were calculated and reported in the results.

(1) $$\text{Casson}\;\;\text{model:}\;\sqrt{\tau }=\sqrt{\tau_{0}}+\sqrt{\eta_{p1}}\cdot \sqrt{\dot{\gamma }}$$

Where τ is shear stress (Pa) is, $\dot{\gamma }$ is shear rate (s^−1^), $\tau_{0}$ is Casson yield value (Pa) and *η*_*p*1_ is Casson plastic viscosity (Pa.s).

### 2.5 Preparation of snacks

The cores were coated with hydrocolloids solution which took 10 s. This process was followed by keeping the samples at ambient temperature for 24 h. After drying of the coated cores, chocolate coating was performed using melted chocolate. Among four sugar-free chocolate formulations, the optimized formulation was selected for the development of chocolate coating based on rheological properties. Four types of snacks were produced as control and main samples: D: snack core coated with chocolate and without hydrocolloids coating; CNT: Core only; DX1,2,3: snack core coated with xanthan gum (1: 0.25%; 2: 0.39%; 3: 0.54%) and chocolate; DP1,2,3: snack core coated with Persian gum (1: 1.5%; 2: 2%; 3: 2.5%) and chocolate.

#### 2.5.1 Water activity, thickness of coating and textural analysis

Water activity analyzer was applied to determine the water activity of the final snack (Lab Master Standard, Novasina, Swiss). Mechanical compression test was conducted using texture analyzer (TA.XTPlus, Stable Micro System, England). During compression tests, the sample holder was kept in a fixed position. The samples were then compressed by the cylindrical probe with 20 mm of diameter, at a constant rate of 0.5 mm/s. The maximum peak positive force required for 40% compaction was determined as hardness and peak negative force as adhesiveness. The thickness of the coatings was measured by image processing with the OCA camera (20, Dataphysics, Germany).

#### 2.5.2 Sensory analysis

Sensory evaluation was carried out at Research Institute of Food Science and Technology (RIFST) preceded by approval of the “RIFST research committee” considering ethical regulations as well as in accordance with the international standards (Shiby et al., 2013; Kiumarsi et al., 2020). 35 untrained panelists (16 men and 19 women ages 25–45 years) who were informed about the experiment and confirmed their consent by signing a form were included in the sensory evaluation. Experiments were conducted using a five-point hedonic scale (1 = dislike extremely; 5 = like extremely). Attributes including appearance, flavor, texture, and overall acceptance were taken into account.

### 2.6 Statistical analysis

All the experiments were done in triplicates. The Analyze of variance (ANOVA) was performed using Minitab (Version 16) software to analyze the data, including fig powder properties (three sizes), Casson plastic viscosity and Casson yield value (4 formula) and final snack properties (8 formula). Mean values were considered significantly different when *p* < 0.05 and significant differences between means were compared by Duncan's test.

## 3 Result and discussion

### 3.1 Fig powders properties

Three types of fig powder with maximum sizes of 707, 505, 354 μm were prepared. Table 2 shows the physicochemical properties of the mentioned powders. Results indicate that there were no significant differences among fig powders having different particle sizes with regard to water activity, moisture content, pH and ash content (*p* < 0.05). Sticky point temperature of the powdered fig could determine the processing condition such as maximum operating temperature range in case of further processing. By increasing the temperature, the fig powder transformed from glassy state to the rubbery phase and became sticky. This temperature is characteristic for each material and is influenced by its glass transition temperature (*T*_*g*_). The stickiness of material is strongly related to the moisture content, due to plasticizering role of water which depresses the *T*_*g*_. Stickiness can be controlled if the temperature of the material is kept below *T*_*g*_. Results indicated that stickiness of all the fig powders occurred at 45 °C. Consequently, the processing conditions and coating temperature should not exceed 45 °C. Compactability of powders however, is defined by the reduction of volume under specified force applied to the powder system. Results showed that the compaction force decreased with the reduction in particle size (*p* < 0.05). Klevan et al. (2010) and Prakash et al. (2011) reported the same results in compressing of drug and fruit powders, respectively. According to their results, following the reduction in particle size, the lower compaction force was required for deformation. When powder compresses, the gas phase is decreased by the approaching of particles to each other. Strong bonds between particles make a compressed sticky mass, which could be considered as a temporary (elastic) or permanent (plastic) deformation. At the present study, three particle sizes of the powder were similar to each other in some physicochemical properties except for required compaction force. Consequently, the powder with the lowest particle size, which had the lowest compaction force, was selected as the core in making the snack.

**Table 2:** Fig powders physicochemical properties.

Experiments	FP 707	FP 505	FP 354
Water activity	0.34 ± 0.01^a^	0.35 ± 0.01^a^	0.35 ± 0.01^a^
Moisture content (%)	10.12 ± 0.07^a^	10.11 ± 0.05^a^	10.11 ± 0.07^a^
pH	4.76 ± 0.015^a^	4.75 ± 0.09^a^	4.76 ± 0.012^a^
Ash content (%)	0.63 ± 0.05^a^	0.61 ± 0.04^a^	0.60 ± 0.04^a^
Sticky point temperature (°C)	45^a^	45^a^	45^a^
Compression force (gf)	2100 ± 35^a^	1823 ± 45^b^	1561 ± 45^c^

FP: Fig Powder; 707, 505 and 354: different particle size of fig powder based on micrometer-sized particles. Different letters (a, b, c) in the same row indicate significant differences (p < 0.05).

### 3.2 Particle size distribution and rheological properties of chocolate

**Figure 1:**
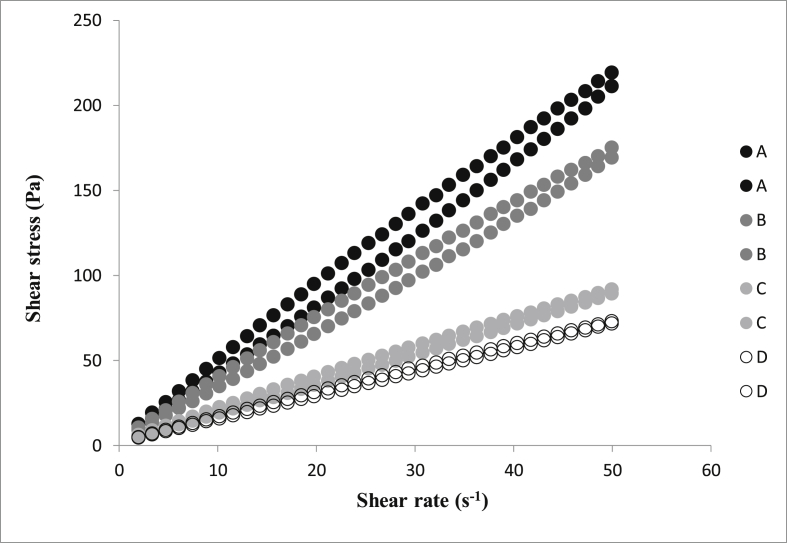
Flow curves (ramp up and ramp down) for chocolate formulations, showing shear stress versus shear rate.: Sample A (black circle), sample B (gray circle), sample C (light gray circle) and sample D (empty circle).

**Figure 2:**
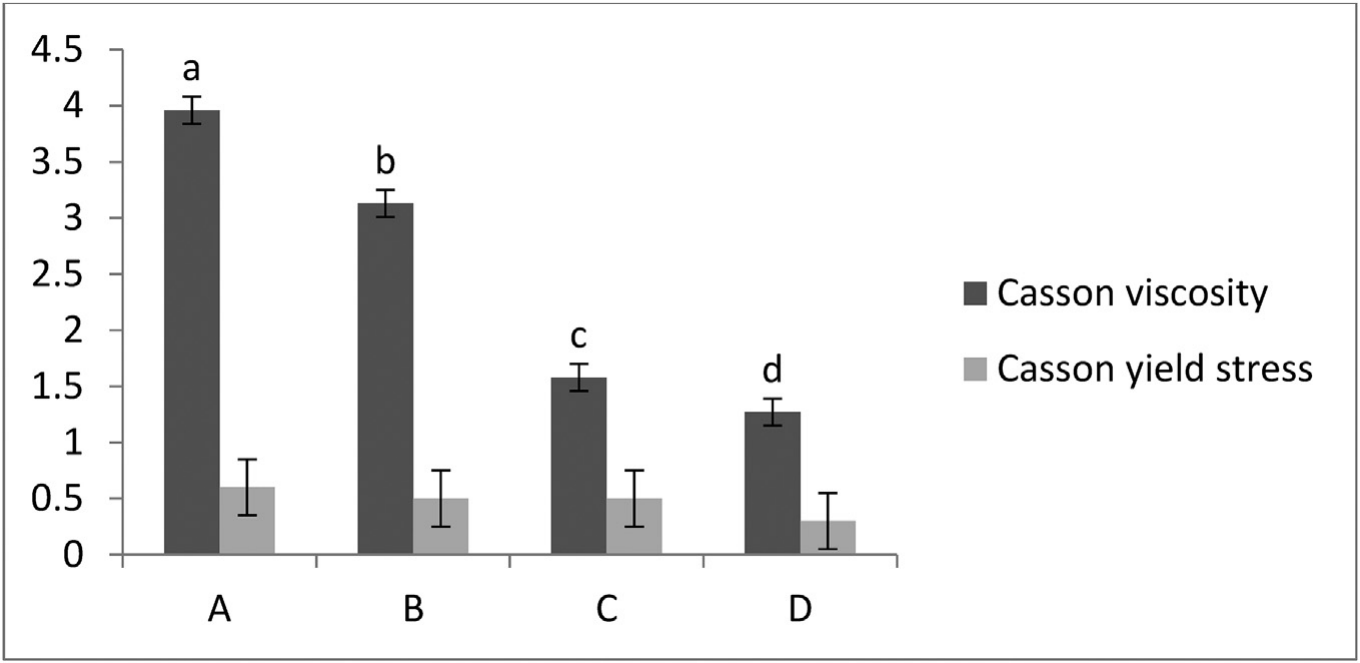
Mean values of Casson plastic viscosity (Pa.s) and Casson yield value (Pa) for prepared chocolate formulations (A, B, C, D). Different letters (a, b, c) indicate significant differences (p < 0.05).

Particle size plays an essential role in chocolate properties. Analysis of the particle size distribution showed that the D_90_ values of all chocolates were lower than 23 μm. Extra fat (from 35 to 40 %) was added at the conching step; hence the particle size was not affected by the fat. Pleasant mouth feeling is expected if the D_90_ values are less than 23 μm, and this has been evaluated to be desirable (Beckett, 2009). Appropriate rheological properties have a crucial role for appropriate coating properties of chocolate (Aeschlimann and Beckett, 2000). Figure 1 presents the flow curves of chocolates showing the values of shear stress as function of shear rate and demonstrates the non-Newtonian behavior of all the samples. Casson viscosity and Casson yield value were calculated using the Casson model by plotting the square root of shear rate against the square root of shear stress. The Casson yield value is calculated as the square of the intercept, and the Casson plastic viscosity as the square of the slope (Abbasi and Farzanmehr, 2009). As Figure 2 presents, no significant difference was observed in Casson yield value of chocolates. However, enhancement of the fat content in chocolate formulations decreased the Casson viscosity significantly (*p* < 0.05). Ghorbel et al. (2011) used chocolate with 45.26–57.05 % fat content for coating of ice cream. They determined the viscosity and yield stress (at 40 °C) between 0.3-0.6 Pa.s and 1–3 Pa, respectively. Coating chocolate with Casson viscosity of 0.2–0.8 Pa.s and Casson yield value of 0.5–3 Pa was also reported by Talbot (1999). The addition of fat content in chocolate formulation, creates some free fat which is called “wetting fat” and could help the particles' movements. This fat could decrease the plastic viscosity by attaching to the surface of the solid particles (Beckett, 2009). Actually, extra fat content could result in the reduction of solid particles concentration and hydrodynamic forces which subsequently decreases the viscosity. It has to be mentioned that the hysteresis happened at the shear stress-shear rate back and forth curves for all of the samples. The presence of hysteresis might be a sign of thixotropic property. Polar interactions with short domain retain the sugar particles, milk powder and solid cocoa particles in connection to each other at the rest time. When the shear stress is applied to the chocolate, these accumulations might break and disappear and this could decrease the viscosity (Servais et al., 2004). Sample D was determined as an appropriate coating formulation for the snack due to the lowest Casson viscosity and Casson yield value.

### 3.3 Snack analysis

#### 3.3.1 Water activity

The results of water activity are shown in Table 3. Hydrocolloid coating over the core can act as a protecting layer against moisture entrance, fat migration and microbial contamination (Gounga et al., 2008). This layer can also retain the aromatic components. Data analysis showed that water activity increased in hydrocolloid coated samples and significant differences were observed among samples coated by different levels of Persian gum and xanthan gum (*p* < 0.05), which may be due to differences in water absorption ability of hydrocolloids. Tavera-Quiroz et al. (2015) reported the same result developing green apple baked snacks. These authors used methylcellulose coating for the snacks and reported the higher moisture content for the coated samples in comparison with non-coated snacks.

**Table 3:** Mean values with standard deviation of snack properties.

	CNT	D	DX1	DX2	DX3	DP1	DP2	DP3
Water activity	0.35 ± 0.01^d^	0.33 ± 0.01^d^	0.42 ± 0.01^c^	0.53 ± 0.01^a^	0.50 ± 0.01^b^	0.43 ± 0.01^c^	0.53 ± 0.01^a^	0.47 ± 0.01^b^
Hardness (gf)	14720 ± 48^b^	19549 ± 48^a^	4689 ± 43^b^	4622 ± 59^d^	5399 ± 25^c^	4860 ± 45^c^	5193 ± 48^c^	5041 ± 48^d^
Adhesiveness (gf)	89 ± 3.3^a^	77 ± 5.3^b^	44.7 ± 5.4^c^	24.8 ± 3.9^d^	20.8 ± 3.1^d^	34.9 ± 3.5^a^	22.6 ± 3.5^d^	14.9 ± 3.5^e^
Coating thickness (mm)		0.076 ± 0.004^a^	0.028 ± 0.001^b^	0.022 ± 0.001^c^	0.015 ± 0.005^d^	0.026 ± 0.001^b^	0.020 ± 0.001^c^	0.014 ± 0.005^d^

D: snack core coated with chocolate and without hydrocolloids coating; CNT: Core only; DX1,2,3: snack core coated with xanthan gum (1: 0.25%; 2: 0.39%; 3: 0.54%) and chocolate; DP1,2,3: snack core coated with Persian gum (1: 1.5%; 2: 2%; 3: 2.5%) and chocolate. Different letters (a, b, c, d, e) in the same row indicate significant differences (p < 0.05).

#### 3.3.2 Textural properties

Coating by hydrocolloids revealed a significant effect on textural properties of the samples (*p* < 0.05). As Table 3 shows, the hardness and adhesiveness of the coated samples were significantly lower than the control samples without hydrocolloid coatings (*p* < 0.05). Coating the core by a hydrocolloid layer creates a proper linkage with the chocolate layer and acts as a lubricating agent. Different levels of xanthan gum and Persian gum showed significant effects on hardness and adhesiveness values (*p* < 0.05). Polysaccharide coatings improve the mechanical properties of food samples by impacting the texture integrity. This is especially important in maintaining the integrity in powder based products which have this potential to be damaged under stress and/or pressure. The samples with hydrocolloid coatings had lower adhesiveness than the samples which were prepared only with chocolate coatings. Moreover, adhesiveness of the samples was decreased by the addition of hydrocolloids concentrations. The nature of the fig powder as core remained the main reason of adhesiveness in the samples. As reported, when the hydrocolloid concentration increases, the adhesion between the sample and the surface of the probe reduces (Banasaz et al., 2013). Our findings indicated a high correlation between adhesiveness and coating layer thickness (r = 0.97) as well as a high negative correlation that was observed between hardness and water activity (r = -0.83).

#### 3.3.3 Coating layer thickness

Chocolate formulations were made up of isomalt sugar and the coating thickness has to be noted since there are limitation on the daily consumption for this sugar alcohol which should not be higher than 20 g (Kruger, 1999). Statistical analysis showed that using of gums as a middle layer between the core and the chocolate decreased the chocolate coating thickness. It was found that the higher the concentration of the gum, the lower was the thickness of coating (*p* < 0.05); while the impact of Persian gum on this thickness reduction was more than xanthan gum. The highest coating thickness was obtained for the sample without hydrocolloid coating and the lowest thickness for the snack with 2.5% Persian gum. Wichchukit et al. (2005) coated a model system with milky chocolate and measured the coating thickness by gravimetric methods. They reported that the coating thickness reduced by the increase in fat content which was due to reduction in viscosity.

#### 3.3.4 Sensory properties

Sensory properties of the samples were evaluated in terms of appearance, flavor, texture, and total acceptance (Table 4). In all attributes, significant differences were observed between samples with and without coating (both chocolate and hydrocolloid) (p < 0.05). Samples coated with chocolate scored higher than the core sample, indicating that panelists liked chocolate appearance more than fig appearance. Coating can greatly influence the organoleptic properties (Yu and MacGregor, 2003). More uniformity in color and thickness is typical in samples with hydrocolloid coating and this could also influence the organoleptic properties (Pajin et al., 2006). In terms of flavor, no significant differences were found among snacks pre-coated by hydrocolloids and chocolate outer shell (p > 0.05). Samples D and CNT received the lowest flavor score which may be due to the role of hydrocolloids in flavor release and better realization of the flavor by the panelists. Regardless of sample DX1 which scored the lowest in texture among samples coated with hydrocolloids, no significant difference was observed between the referred coated samples. The low score by panelists may be due to the higher adhesiveness and/or hardness of this treatment (Table 4). Total acceptance significantly increased with the elevated concentration of hydrocolloid coating; therefore, snack with 1.5% Persian gum obtained the highest score (Table 4). Gounga et al. (2008) used a whey protein isolate-pullulan layer coating between chocolate layer and dried chestnut in the making of a chocolate snack based on chestnut. They reported that WPI improved chocolate coating and sensory properties. According to their reports, recoating of the samples with chocolate increased both its total acceptance and its shelf life.

**Table 4:** Sensory properties of snack.

Samples	Appearance	Flavor	Texture	Total acceptance
DX1	4.3 ± 0.27^a^	4.25 ± 0.24^a^	3.8 ± 0.31^b^	4.05 ± 0.19^ab^
DX2	4.25 ± 0.25^a^	4.2 ± 0.22^a^	4.1 ± 0.25^a^	4.25 ± 0.23^a^
DX3	4.25 ± 0.25^a^	4.1 ± 0.23^a^	4 ± 0.12^a^	4.15 ± 0.29^a^
DP1	4.27 ± 0.13^a^	4.2 ± 0.24^a^	4.15 ± 0.27^a^	4.2 ± 0.22^a^
DP2	4.15 ± 0.27^a^	4 ± 0.24^a^	4.3 ± 0.27^a^	4.15 ± 0.26^a^
DP3	4.23 ± 0.25^a^	4.15 ± 0.28^a^	4.35 ± 0.28^a^	4.2 ± 0.21^a^
D	4.02 ± 0.21^b^	3 ± 0.15^b^	3.1 ± 0.11^bc^	3.25 ± 0.14^bc^
CNT	3.45 ± 0.25^c^	2.8 ± 0.14^b^	2.35 ± 0.28^c^	2.7 ± 0.17^c^

D: snack core coated with chocolate and without hydrocolloids coating; CNT: Core only; DX1, 2,3: snack core coated with xanthan gum (1: 0.25%; 2: 0.39%; 3: 0.54%) and chocolate; DP1,2,3: snack core coated with Persian gum (1: 1.5%; 2: 2%; 3: 2.5%) and chocolate. Different letters (a, b, c) in the same column indicate significant differences (p < 0.05).

## 4 Conclusion

A healthier formulation for the snack foods is of utmost importance due to the effect on their quality. Sometimes, in a number of snacks, only tiny changes in the formulations can improve the functional and health properties enormously. At the present study, a novel snack based on fig fruit powder was developed. Fig powder as the core, hydrocolloid solution as the middle layer and chocolate layer as the outer shell were used to make the entitled snack. Chocolate properties were a critical parameter for making this snack. If inappropriate chocolate is used, fat migration and recrystallization occurs in case of a change in the surrounding temperature. Chocolate containing 29.3% isomalt, 0.7% lecithin, 40% cocoa butter and 30% cocoa powder was preferred to be used for the coating of the snack. Coating of the core in the middle layer was performed with Persian gum and xanthan gum in three concentrations. Furthermore the examination of the hardness and adhesiveness of the coated samples showed that the ones with hydrocolloids had lower hardness and adhesiveness in comparison to the control and that the increase in hydrocolloids concentration resulted in lower thickness for the chocolate coating. Using hydrocolloids met the aim of improving sensory properties and textural characteristics. Moreover it served the goal of a better chocolate recoating. Selecting a fig powder with the maximum particle size of 354 μm, application of Persian gum with a concentration of 2.5% and the chocolate with suitable rheological properties were the main key factors in formulation development of a novel functional fruit snack based on fig fruit and coated with sugar-free chocolate.

## Declarations

### Author contribution statement

Samira Yeganehzad, Mansour R. Ashkezary: Conceived and designed the experiments; Analyzed and interpreted the data; Wrote the paper.

Maryam Kiumarsi: Performed the experiments; Analyzed and interpreted the data; Contributed reagents, materials, analysis tools or data.

Narjes Nadali: Performed the experiments; Analyzed and interpreted the data; Contributed reagents, materials, analysis tools or data; Wrote the paper.

### Funding statement

This research did not receive any specific grant from funding agencies in the public, commercial, or not-for-profit sectors.

### Competing interest statement

The authors declare no conflict of interest.

### Additional information

No additional information is available for this paper.
